# Bilateral Simultaneous Asymmetrical Anterior Shoulder Dislocation With a Fracture

**DOI:** 10.7759/cureus.16783

**Published:** 2021-07-31

**Authors:** Yousef Al-Khatib, Muhammad Adeel Akhtar, Ata Kasis

**Affiliations:** 1 Orthopaedics, Royal College of Surgeons in Ireland, Dublin, IRL; 2 Trauma and Orthopaedics, Royal College of Surgeons in Ireland, Dublin, IRL; 3 Trauma and Orthopaedics, Wansbeck General Hospital, Ashington, GBR

**Keywords:** bilateral shoulder dislocation, hill sachs lesion, avulsion humeral fracture, orthopaedics, trauma and orthopaedics, anterior shoulder dislocation

## Abstract

In this case report, we present the case of a 19-year-old male who presented to the emergency department with bilateral shoulder pain and significantly decreased range of motion. His X-rays showed a bilateral dislocation of the glenohumeral joints, along with a proximal humeral fracture. Shoulder dislocations were manipulated, while the proximal humeral fracture was conservatively managed. Bilateral simultaneous asymmetric anterior shoulder dislocations are uncommon in such a young age group, especially when associated with a sports injury.

## Introduction

Shoulder dislocations are common and frequently encountered in the emergency department. Unilateral anterior dislocations are the most common and account for 95% of the presentations. In contrast, bilateral dislocations are uncommon, with bilateral anterior dislocations being even rarer than posterior dislocations [1]. Here, we report the case of a 19-year-old male who presented with a bilateral asymmetrical anterior shoulder dislocation with an avulsion fracture of the left greater tuberosity.

## Case presentation

A 19-year-old male was brought to the emergency department with severe pain and significantly decreased range of motion in the shoulders bilaterally after colliding with another individual during a football match. Apart from a high body mass index, the patient was otherwise healthy with no comorbidities. There was no significant family or social history, and he had no history of allergies.

Examination

On examination, the patient’s vital signs were stable. His radial pulses were palpable and both sharp and soft sensations were intact bilaterally. There was a visible shoulder deformity with tenderness on palpation, while the movement of the glenohumeral joint elicited extreme pain bilaterally. There was a significantly decreased range of motion in the shoulders bilaterally.

Diagnostic imaging

**Figure 1:**
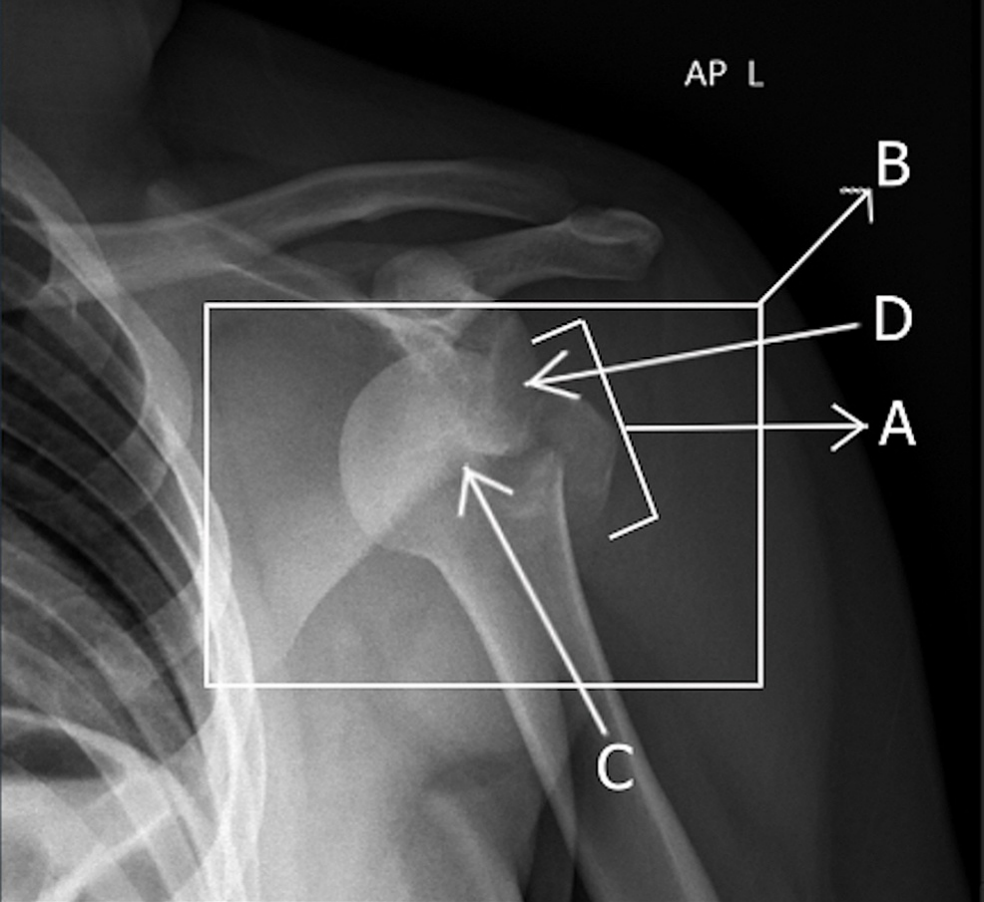
Anterior-posterior X-ray showing the anterior left glenohumeral joint dislocation with an avulsion fracture of the greater tuberosity. The avulsion fracture of the greater tuberosity (A), and the anterior left glenohumeral joint dislocation (B) can be seen. The humeral head (C) is dislocated in an anterior direction to the glenoid (D).

**Figure 2:**
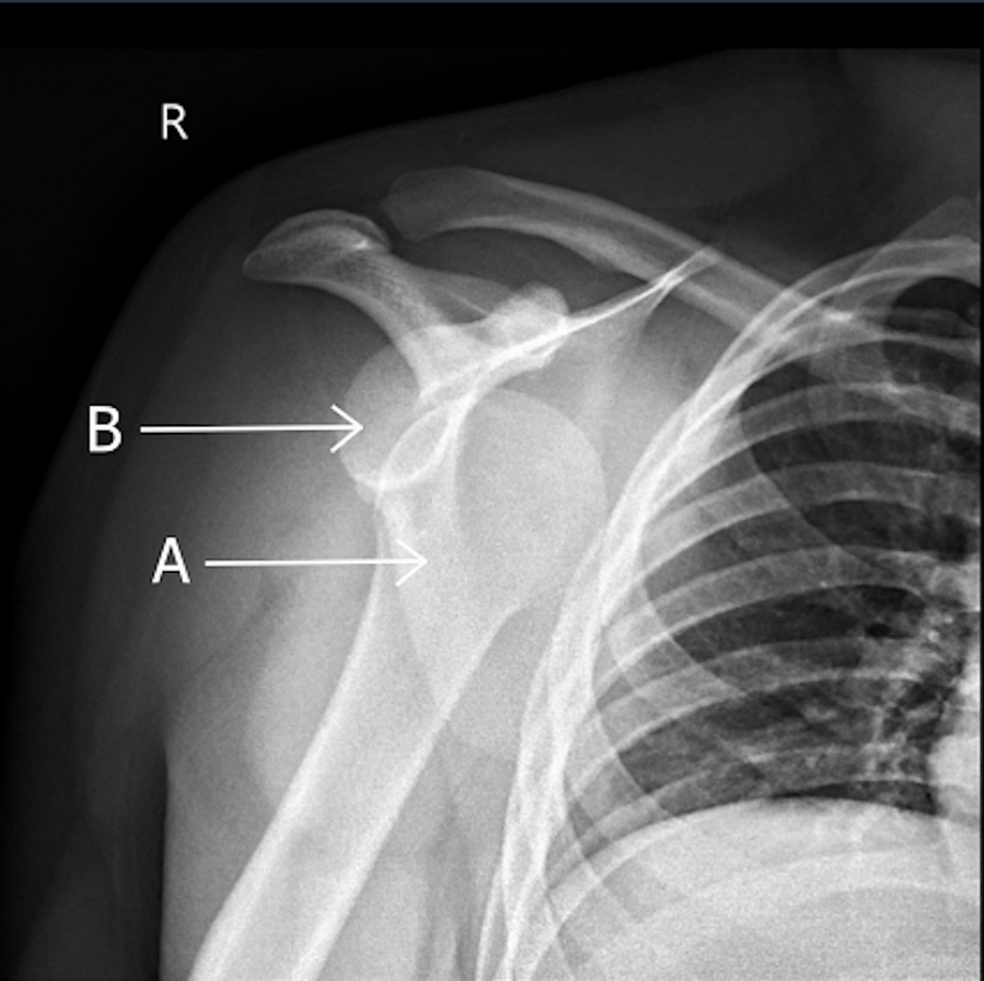
Anterior-posterior X-ray showing the anteriorly dislocated right glenohumeral joint. The head of the humerus (A) is dislocated anterior to the glenoid (B).

X-ray images demonstrated an asymmetrical dislocation of both glenohumeral joints with an avulsion fracture of the greater tuberosity of the left humerus (Figures 1, 2).

Intervention

Both dislocations were subsequently reduced using the Hippocratic manoeuvre under sedation using pentothal, and the patient decided to treat his humeral fracture conservatively. Subsequently, strict immobilisation was applied with a body bandage brace on both arms for four weeks, followed by a sling for two weeks. In addition, the patient was referred for physiotherapy which he was not compliant with.

Follow-up

**Figure 3:**
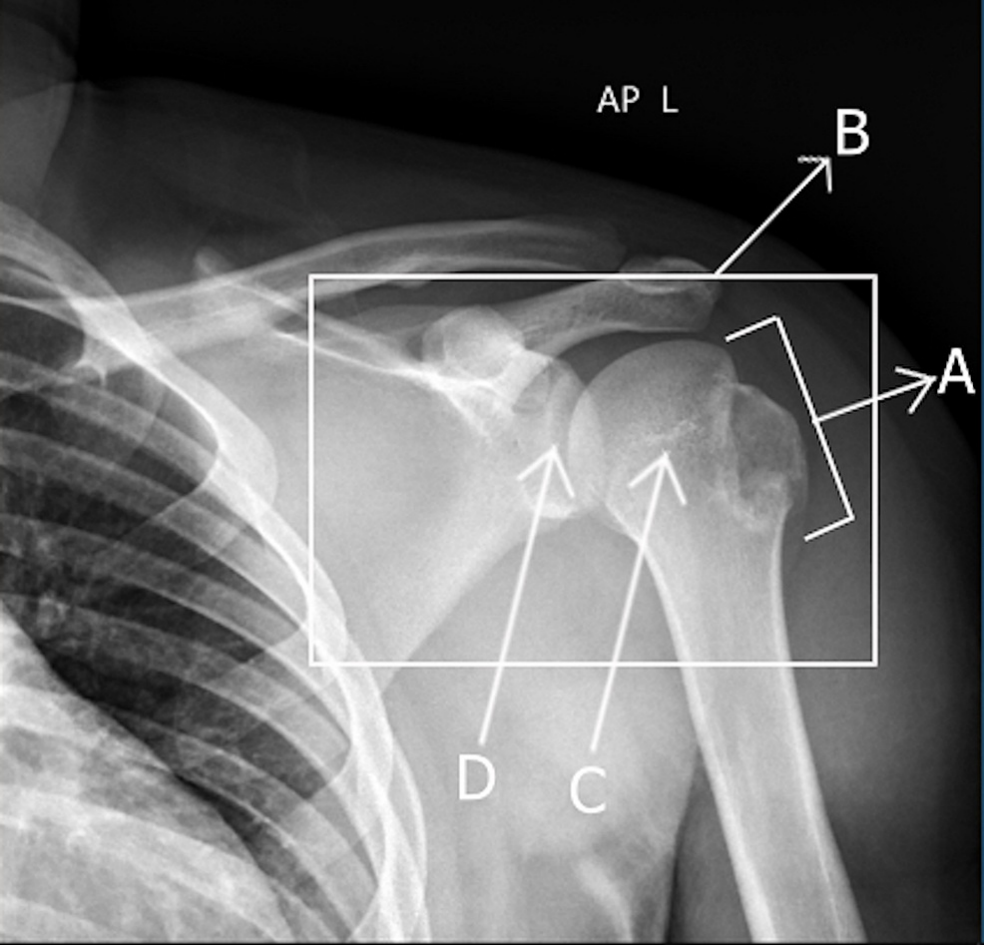
Anterior-posterior X-ray showing the left glenohumeral joint post-reduction with the avulsion fracture of the greater tuberosity still visible. The avulsion fracture of the greater tuberosity is still visible (A), and the left glenohumeral joint post-reduction (B) can be seen. The humeral head (C) is back in the socket articulating with the glenoid (D).

**Figure 4:**
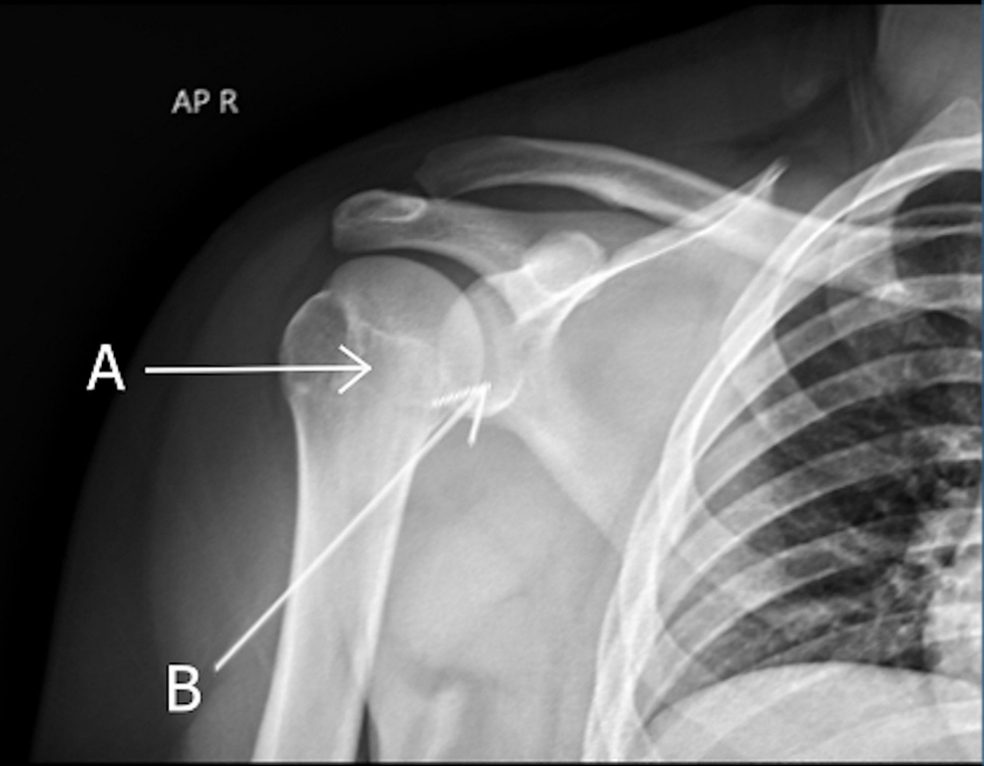
Anterior-posterior X-ray showing the right glenohumeral joint post-reduction. The humeral head (A) is back in the socket and articulating with the glenoid (B).

Six weeks post-reduction, X-ray images confirmed that the two dislocations were reduced (Figures 3, 4). The fracture at the greater tuberosity was still evident along with some lucency around the humeral bladder.

During a telephonic follow-up 18 months post-reduction, the patient reported slight shoulder pain with no further dislocations.

## Discussion

Shoulder dislocations are a common emergency encountered by orthopaedic surgeons [2]. Of note, 76% of shoulder dislocations occur in males and account for 85% of all dislocations [3-6]. These dislocations can be classified as being unilateral or bilateral, while the direction of dislocation can be anterior, posterior, or inferior. Overall, 95% of these injuries are unilateral with an anterior displacement, with posterior unilateral dislocations accounting for less than 5% of the cases [1,3,7]. Bilateral shoulder dislocations are even less common, most commonly occurring in the posterior direction often after a seizure [1]. Anterior dislocations usually occur during sports injuries which result in forced extension, abduction, and external rotation of the shoulder joint, whereas posterior dislocations often occur following axial loading on an arm that is adducted, flexed, and internally rotated [7]. Because there is a 52% recurrence rate of shoulder dislocations in younger populations, it is important to ensure that strict immobilisation is applied [7,8]. Regarding sports injuries, another consideration needs to be the time before return to play to prevent further dislocations [7]. Currently, there is no consensus on a time, but return to play can occur two to three weeks post-reduction. However, the decision should be individualized to each patient after a thorough clinical examination as the recurrence rate varies between 37% and 90% with a short time before return to play [7].

After conducting a literature search, we found several reports of bilateral anterior shoulder dislocations. However, these were mainly following seizure activity and a few with an associated Hill-Sachs lesion [9-11]. Our case is a rare presentation of bilateral anterior shoulder dislocation because it occurred following a low-intensity collision with an associated greater tuberosity fracture, which occurs in 15% of bilateral anterior dislocations and is more common in patients older than 40 years of age [4].

To our knowledge, there is only one previous case of bilateral anterior shoulder dislocation which occurred without an obvious cause and one case of bilateral shoulder dislocation with a unilateral fracture [12,13]. Our case is an important addition to the literature of bilateral anterior shoulder dislocations as it is only the second case which occurred after a minimum impact in an adolescent and the only case where a simultaneous anterior bilateral glenohumeral joint dislocation had a unilateral proximal humeral fracture.

To prevent such atypical presentations from being overlooked or their associated injuries, MRI should be used for diagnosis which can aid in identifying associated fractures or rotator cuff tears while also ensuring congruent reduction of the shoulder and neurovascular status [14]. Similarly, X-ray images for both shoulders should be obtained to achieve an accurate diagnosis and prevent bilateral dislocations from being missed. Following reduction, special consideration should be given to regular follow-up and further treatment of any instability to reduce the risk of recurrence.

## Conclusions

We present a case of bilateral anterior shoulder dislocation with an associated greater tuberosity fracture after a minimal impact collision. This serves as a reminder to keep bilateral anterior shoulder dislocation on the differential even when there is no seizure activity, high-impact trauma, or electrocution in the patient history. Finally, we recommend considering the use of MRI in the acute phase to detect soft tissue injuries when there is a high clinical suspicion.

## References

[1] Lasanianos N, Mouzopoulos G (2008). An undiagnosed bilateral anterior shoulder dislocation after a seizure: a case report. Cases J.

[2] Auerbach B, Bitterman A, Mathew C, Healy W 3rd (2015). Bilateral shoulder dislocation presenting as a unilateral shoulder dislocation: case report. J Am Osteopath Assoc.

[3] Cutts S, Prempeh M, Drew S (2009). Anterior shoulder dislocation. Ann R Coll Surg Engl.

[4] Shah A, Judge A, Delmestri A (2017). Incidence of shoulder dislocations in the UK, 1995-2015: a population-based cohort study. BMJ Open.

[5] Kalkan T, Demirkale I, Ocguder A, Unlu S, Bozkurt M (2009). [Bilateral anterior shoulder dislocation in two cases due to housework accidents]. Acta Orthop Traumatol Turc.

[6] O'connor-Read L, Bloch B, Brownlow H (2007). A missed orthopaedic injury following a seizure: a case report. J Med Case Rep.

[7] Sofu H, Gürsu S, Koçkara N, Öner A, Issın A, Çamurcu Y (2014). Recurrent anterior shoulder instability: review of the literature and current concepts. World J Clin Cases.

[8] Kokkalis ZT, Iliopoulos ID, Antoniou G, Antoniadou T, Mavrogenis AF, Panagiotopoulos E (2017). Posterior shoulder fracture-dislocation: an update with treatment algorithm. Eur J Orthop Surg Traumatol.

[9] Sharma L, Pankaj A, Kumar V, Malhotra R, Bhan S (2005). Bilateral anterior dislocation of the shoulders with proximal humeral fractures: a case report. J Orthop Surg (Hong Kong).

[10] Ozçelik A, Dinçer M, Cetinkanat H (2006). Recurrent bilateral dislocation of the shoulders due to nocturnal hypoglycemia: a case report. Diabetes Res Clin Pract.

[11] Sachit M, Shekhar A, Shekhar S, Joban SH (2015). Acute spontaneous atraumatic bilateral anterior dislocation of the shoulder joint with Hill-Sach’s lesions: a rare case. J Orthop Case Rep.

[12] Bellazzini MA, Deming DA (2007). Bilateral anterior shoulder dislocation in a young and healthy man without obvious cause. Am J Emerg Med.

[13] Sharma D, M K, R NA, Poduval M, Patro DK (2013). Asymmetrical fracture dislocation of shoulder - a case report and review of literature. J Orthop Case Rep.

[14] Taneja AK, Pecci Neto L, Skaf A (2013). Bilateral anterior glenohumeral dislocation and coracoid processes fracture after seizure: acute MRI findings of this rare association. Clin Imaging.

